# Hippocampal volume and volume asymmetry prospectively predict PTSD symptom emergence among Iraq-deployed soldiers

**DOI:** 10.1017/S0033291721003548

**Published:** 2023-04

**Authors:** Adam R. Cobb, Mikael Rubin, Deborah L. Stote, Brian C. Baldwin, Han-Joo Lee, Ahmad R. Hariri, Michael J. Telch

**Affiliations:** 1 Department of Psychology, The University of Texas at Austin, Austin, TX, USA; 2 Department of Psychiatry and Behavioral Sciences, Medical University of South Carolina, Charleston, SC, USA; 3 PTSD Clinical Team, Ralph H. Johnson VA Medical Center, Charleston, SC, USA; 4 Department of Psychology, University of Wisconsin-Milwaukee, Milwaukee, WI, USA; 5 Department of Psychology and Neuroscience, Duke University, Durham, NC, USA

**Keywords:** PTSD, hippocampal volume, military, prospective, war-zone

## Abstract

**Background:**

Evidence suggests a link between smaller hippocampal volume (HV) and post-traumatic stress disorder (PTSD). However, there has been little prospective research testing this question directly and it remains unclear whether smaller HV confers risk or is a consequence of traumatization and PTSD.

**Methods:**

U.S. soldiers (*N* = 107) completed a battery of clinical assessments, including structural magnetic resonance imaging pre-deployment. Once deployed they completed monthly assessments of traumatic-stressors and symptoms. We hypothesized that smaller HV would potentiate the effects of traumatic stressors on PTSD symptoms in theater. Analyses evaluated whether total HV, lateral (right *v.* left) HV, or HV asymmetry (right – left) moderated the effects of stressor-exposure during deployment on PTSD symptoms.

**Results:**

Findings revealed no interaction between total HV and average monthly traumatic-stressors on PTSD symptoms *b* = −0.028, *p* = 0.681 [95% confidence interval (CI) −0.167 to 0.100]. However, in the context of greater exposure to average monthly traumatic stressors, greater right HV was associated with fewer PTSD symptoms *b* = −0.467, *p* = 0.023 (95% CI −0.786 to −0.013), whereas greater left HV was unexpectedly associated with greater PTSD symptoms *b* = 0.435, *p* = 0.024 (95% CI 0.028–0.715).

**Conclusions:**

Our findings highlight the importance of considering the complex role of HV, in particular HV asymmetry, in predicting the emergence of PTSD symptoms in response to war-zone trauma.

## Introduction

The hippocampus is a primary brain region implicated in neurobiological models of post-traumatic stress disorder (PTSD) (Pitman et al., 2012), and there is extensive evidence for smaller hippocampal volume (HV) in PTSD (Karl et al., 2006; Logue et al., 2018; O'Doherty, Chitty, Saddiqui, Bennett, & Lagopoulos, 2015; Smith, 2005; Woon, Sood, & Hedges, 2010). Smaller HV has been linked with PTSD following trauma exposure (Gilbertson et al., 2002; Apfel et al., 2011), whereas greater baseline HV has been shown to predict a favorable response to trauma-focused cognitive-behavioral therapy (Rubin et al., 2016; van Rooij et al., 2016). A common explanation for the link between smaller HV and PTSD is that trauma exposure causes HV reductions, which is plausible, given established evidence for stress-evoked glucocorticoid-mediated hippocampal atrophy (Sapolsky, 2000). An alternative, but not mutually exclusive explanation is supported by evidence suggesting smaller HV may potentially operate as a pre-existing risk factor (e.g. Gilbertson et al., 2002). However, a lack of prospective studies has made it difficult to address the possibility that smaller HV confers risk as well and is not merely a consequence of trauma or the post-trauma emergence of PTSD symptoms.

Only one prior study has examined HV as a prospective risk factor for PTSD symptoms (Admon et al., 2013). Israeli combat paramedics (*N* = 33) underwent structural brain imaging prior to and 18 months later during military service. Smaller HV was not shown to predict the later emergence of PTSD symptoms, whereas decreases in HV were associated with PTSD symptoms at follow-up. This suggests that instead of smaller HV reflecting pre-trauma susceptibility, reductions in HV may reflect the sequela of trauma or development of PTSD. This conclusion is consistent with the hypothesized contribution of stress-evoked hippocampal atrophy to stress-related psychopathology (Sapolsky, 2000), and prior cross-sectional findings of reduced HV in individuals with PTSD (O'Doherty et al., 2015; Woon et al., 2010).

While existing evidence implies a causal relation between stress-evoked HV reductions and PTSD, a limitation of prior research is a lack of evaluating pathways through which reduced HV may cause PTSD. Diathesis-stress models provide more complete functional accounts of how individual differences moderate the impact of stress. These frameworks have been used to evaluate the risk for several forms of stress-related psychopathology and other adverse outcomes (Edmondson et al., 2014; Elwood, Hahn, Olatunji, & Williams, 2009), but have not been used to identify pre-trauma neurological factors that predict the development of PTSD. The key advantage of a diathesis-stress approach is the potential to inform how smaller HV might functionally relate to the development of PTSD symptoms. If smaller HV operates as a pre-trauma susceptibility factor, it should be expected to moderate the impact of traumatic stress.

The current investigation is one of a series from the Texas Combat Stress Risk Project, which aims to identify biological, cognitive, and psychosocial risk factors for war-zone stress-evoked psychopathology (Beevers, Lee, Wells, Ellis, & Telch, 2011a; Beevers et al., 2011b; Cobb, Josephs, Lancaster, Lee, & Telch, 2018, 2017; Disner et al., 2013; Josephs, Cobb, Lancaster, Lee, & Telch, 2017; Lancaster, Cobb, Lee, & Telch, 2016; Lee, Goudarzi, Baldwin, Rosenfield, & Telch, 2011; Telch et al., 2015, 2012) An exploratory analysis probed the effect of hippocampal asymmetry (the difference between left and right HV) as a moderator of war-zone stressors impact on the emergence of PTSD symptoms during war-zone deployment.

## Methods and materials

### Participants

Soldiers (*N* = 107) from the parent project were recruited from nine Army units (four combat service support units, four combat units, and one combat support unit) deployed from Ft. Hood to Iraq between August 2007 and August 2009. Unit leaders were absent from recruitment and briefing, which was provided by the PI (MJ Telch) and the project manager (BC Baldwin) in the presence of an ombudsman to ensure soldiers felt free to choose whether to participate. Participants were (1) ⩾ 18 years of age, with (2) no prior deployments, and (3) planned deployment within 3 months of study enrollment.

Of the 223 soldiers who attended the briefings, 184 consented to participate, six did not deploy, and one withdrew consent. Of the 177 remaining, 32 did not complete the neuroimaging assessment, 17 (13%) were excluded due to non-viable structural scans, and 21 did not complete in-theater assessments. Among the 107 included in the final sample, participants completed an average of 10.88 monthly in-theater assessments, with a range of deployment durations from 2 to 21 months (see Table 1 for demographics and descriptive statistics). There were no apparent patterns to missing data, including with respect to completing neuroimaging and in-theater assessments.

**Table 1.** Descriptive statistics

Variable	*N*	%			
Lifetime axis I diagnosis	58.00	54.21			
Male	93.00	86.92			
Female	14.00	13.08			
Prior trauma exposure	26.00	24.30			
Threshold for PTSD (SPRINT > 14)	30.00	28.04			
Variable	*N*	*M*	s.d.	*Min*	*Max*
Age	107	23.31	5.39	18.04	43.11
Monthly CEL entries	107	10.88	4.88	2.00	21.00
Monthly average PTEs	107	1.92	1.98	0.00	10.00
Trait anxiety (STAI)	107	37.19	9.00	20.00	62.00
In-theater depression (CES-D-10)	107	7.82	4.60	0.00	30.00
In-theater PTSD symptoms (SPRINT)	107	6.06	7.05	0.00	40.00
L HV	107	4526.10	459.79	3402.00	57 589.00
R HV	107	4776.81	433.01	3688.00	5999.00

*Note.* Table 1 presents descriptive statistics for all modeled variables. Sex (male = 0) and Axis I psychopathology (0 = absent) were entered as dichotomous predictors, whereas all other predictors were *z*-transformed. CEL, Combat experiences log, completed monthly in theater; PTEs, potentially traumatic events; STAI, State-Trait Anxiety Inventory; CES-D-10, Center for Epidemiological Studies Depression Scale (10 items), administered monthly during deployment; SPRINT, Short Post-Traumatic Stress Disorder Rating Interview), administered monthly during deployment. L HV and R HV, left and right hippocampal volume (cm3), adjusted for total intracranial volume (ICV; cm^3^). R – L HV, right – left hippocampal volume. The threshold for soldiers that met possible PTSD at any point in their deployment reflects the standard cutoff for the SPRINT and was derived by calculating the maximum SPRINT score endorsed at any point during deployment.

### Procedure

All study procedures were approved by the IRBs at Brooke Army Medical Center at Fort Sam Houston and the University of Texas at Austin. Prior to their deployment, soldiers completed an extensive pre-deployment assessment spanning biological, cognitive, and psychosocial domains, and a structured diagnostic interview at the University of Texas at Austin (see Lee et al., 2011 for details). Once deployed to Iraq, soldiers received monthly email reminders prompting their completion of the Combat Experiences Log (19), which assessed the type and severity of war-zone stressors and stress-related symptoms during the previous month.

### Pre-deployment assessment measures

Demographics, Trauma Exposure, and Lifetime Psychopathology. At pre-deployment, all soldiers provided demographics, and were assessed using the Structured Clinical Interview for DSM-IV Axis I Disorders (SCID-I-IV) by advanced doctoral students with > 1 year of assessment experience, supervised by the PI (MJ Telch). The SCID-I-IV was used to assess past and current psychopathology, and past exposure to trauma as defined by PTSD Criterion A.

Trait Anxiety. Trait anxiety was assessed with the State-Trait Anxiety Inventory (Spielberger, Gorsuch, & Lushene, 1970). The STAI-T indexes the tendency to experience anxiety in multiple contexts, with items rated by frequency (0 = ‘Almost never**’**; 3 = **‘**Almost always**’**). It has been extensively validated (Bieling, Antony, & Swinson, 1998). Internal consistency for this sample was excellent (*α* = 0.91).

Brain Imaging. Volumetric magnetic resonance imaging data were collected using a GE 3.0T Signa Excite scanner. An automated shim procedure was applied to minimize possible magnetic field inhomogeneities. Two high-resolution T1-weighted SPGR scans optimized for high contrast between gray, white, and CSF on a sagittal plane using 1.3 mm slice thickness (*T* = 5 ms, TE = 1.2 ms, flip angle = 11°) were acquired for morphometric analyses. Cortical reconstruction and volumetric segmentation were performed using the FreeSurfer image analysis suite (version 4.02; http://surfer.nmr.mgh.harvard.edu/). This involved motion correction and averaging of two volumetric T1 weighted images, removal of non-brain tissue, automated Talairach transformation, segmentation of the subcortical white and deep gray matter volumetric structures, intensity normalization, tessellation of the gray−white matter boundary, automated topology correction, and surface deformation following intensity gradients to optimally place gray-white and gray−CSF borders at the location where the greatest shift in intensity defines the transition to the other tissue class. FreeSurfer morphometric procedures have been demonstrated to show good test–retest reliability across scanner manufacturers and field strengths. The primary values of interest from the structural MRI were total HV as well as lateral (left and right) HV. We also explored right – left HV asymmetry. We controlled for total intracranial volume (ICV) by calculating the proportion of total HV and lateral HV relative to ICV.

### Deployment assessment measures

#### The Combat Experiences Log (CEL)

During deployment, soldiers completed monthly self-report measures of stressor exposure and stress-related symptoms using a web-based self-monitoring assessment system (Lee et al., 2011; see below).

#### War-zone stressors

The monthly occurrence of war-zone stressors in the past 30 days was assessed with dichotomous (present *v.* absent) items adapted from the Deployment Risk and Resilience Inventory (King, King, Foy, Keane, & Fairbank, 1999). Because we were interested in the impact of potentially traumatic events (PTEs), two advanced doctoral students and the PI (MJ Telch) independently selected for inclusion the subset of DRRI items that met the DSM-5 criterion A definition for trauma exposure (‘exposure to actual or threatened death, serious injury or sexual violence’, p. 271; American Psychiatric Association, 2013). Additionally, two free-response items that allowed coding of stressors not included in the checklist were independently coded. Raters agreed on 100% of the selected items.

From these items representing in-theater stressor counts, we derived two stressor variables: between-soldier differences in average monthly exposure to traumatic war-zone stressors (PTE_BP_), and within-soldier monthly deviation from this average (PTE_WP_). Separately considering these components avoids the problematic assumption that the effects of traumatic-stressors aggregated over time and acute changes in stressor exposure are equal (Hoffman & Stawski, 2009). Disentangling these components is critical, especially in the context of a longitudinal diathesis-stress framework.

#### In-theater depression symptoms (CES-D-10)

In-theater monthly depression symptoms were assessed using the Center for Epidemiological Studies Depression Scale – 10 item version (Andresen et al., 1994). Items were rated according to frequency of each core symptom of depression (0 = ‘Rarely/None of the time’; 3 = ‘Most/All of the time’) in the past 30 days. The CES-D-10 is well-validated and highly correlated with the full 20-item version. Internal consistency was good in the present sample (*α* = 0.81).

#### In-theater post-traumatic stress symptoms (PTSD symptoms)

In-theater monthly severity of PTSD symptoms was assessed using an expanded self-report version of The Short Post-Traumatic Stress Disorder Interview Scale (SPRINT; Connor & Davidson, 2001; Norris, Hamblen, Brown, & Schinka, 2008). This measure includes 11 Likert-type items rated according to the severity of core PTSD symptoms occurring in the past 30-days, with total monthly scores as our primary outcome of interest. The SPRINT demonstrates excellent psychometric properties and has good convergent validity with related measures. Internal consistency for the present sample was excellent (*α* = 0.90).

### Statistical analyses

To address our primary aims we conducted generalized mixed-effects models using the lme4 package in R (Bates, Mächler, Bolker, & Walker, 2015). We used a maximal approach to model building, with initial selection of the combination of fixed and random effects of time as well as the predictors of interest. All models fit best using fixed and random linear effects of time and were retained. We used the Poisson family to model monthly in-theater PTSD symptoms, and a square-root link function to address non-normal residuals. To handle overdispersion in the model (a common issue with Poisson distributed data), we also included an observation-level random effect, which addressed the overdispersion effectively. We included age, sex (0 = male), current and past Axis I psychopathology (0 = absent), prior trauma exposure (0 = absent), trait anxiety, time since deployment, and monthly in-theater depression symptoms as covariates in all of the models. All HV indices were calculated as a proportion of ICV. All continuous predictors were standardized (scaled and centered), with the exception of time, which was entered in its natural metric, and centered at 9 months (i.e. to stabilize residuals).

To address our primary aims we (1) evaluated the interactions between pre-deployment total HV and between-soldier differences in average monthly exposure to traumatic war-zone stressors (PTE_BP_), and within-soldier monthly deviation from this average (PTE_WP_) and (2) evaluated the distinct interactions between lateral (right and left) HV and PTE_BP_ or PTE_WP_. We then examined HV asymmetry (the right-left HV discrepancy) as the moderator of PTE_BP_ or PTE_WP_ to further probe the findings of our analysis of lateralized HVs. Additionally, in response to reviewer feedback, we replicated all analyses excluding covariates to help clarify the effects of HV and in-theater trauma-exposure on PTSD symptoms. The results of these additional analyses are provided in online Supplementary Tables S3–S5. All continuous predictors were standardized. Each fixed effect estimate was exponentiated to allow for interpretation of coefficients as the change in the outcome given one standardized unit change in the predictor. We do not report random effects from the models. We bootstrapped all models based on profile likelihood with 1000 iterations, using the default methods from the MASS package in R to generate 95% confidence intervals (CIs) around parameter estimates.

## Results

Main effects for all models are reported in the tables (Table 2 and online Supplementary Tables S1–S2), which provide direct effects for HV on PTSD symptoms. Findings revealed no direct effects of pre-deployment HV on in-theater PTSD symptoms across HV indices, including for total HV [*b* = −0.20, *p* = 0.071 (95% CI −0.42 to 0.02)], left HV [*b* = −0.03, *p* = 0.872 (95% CI −0.39 to 0.33)], right HV [*b* = −0.18, *p* = 0.460 (95% CI −0.52 to 0.17)], and right – left HV asymmetry [*b* = −0.08, *p* = 0.460 (95% CI −0.29 to 0.13)].

**Table 2.** Regression models

						*95% CI*	
Main effects	*exp*(*b*)	*b*	s.e.	*z*	*p*	*Lower*	*Upper*
Intercept	6.87	1.93	0.17	11.40	0.000***	1.63	2.26
Months	1.00	0.00	0.02	−0.11	0.914	−0.03	0.03
Sex (male = 0)	1.96	0.68	0.29	2.08	0.038*	0.03	1.25
Age	−0.94	−0.06	0.10	−0.65	0.518	−0.25	0.12
Axis I psychopathology (Absent = 0)	1.50	0.41	0.21	1.95	0.051	0.01	0.81
Prior trauma (Absent = 0)	−0.77	−0.26	0.25	−1.06	0.291	−0.72	0.21
CESD	1.77	0.56	0.08	7.05	0.000***	0.38	0.69
STAI	1.10	0.09	0.10	0.86	0.392	−0.10	0.30
PTE_BP_	1.15	0.05	0.13	0.38	0.706	−0.10	0.38
PTE_WP_	1.16	0.06	0.03	2.05	0.040*	0.03	0.25
Avg. HV	−0.82	−0.20	0.11	−1.80	0.071	−0.40	0.03
Total HV model	*exp*(*b*)	*b*	s.e.	*z*	*p*	*Lower*	*Upper*
Avg. HV* PTE_BP_	−0.98	−0.03	0.14	−0.18	0.858	−0.30	0.22
Avg. HV* PTE_WP_	−0.97	−0.03	0.07	−0.41	0.681	−0.17	0.10
Lateral HV model	*exp*(*b*)	*b*	s.e.	*z*	*p*	*Lower*	*Upper*
L HV* PTE_BP_	1.54	0.43	0.19	2.26	0.024*	0.02	0.71
L HV* PTE_WP_	1.02	0.02	0.10	0.24	0.813	−0.16	0.23
R HV* PTE_BP_	−0.63	−0.47	0.21	−2.27	0.023*	−0.79	−0.01
R HV* PTE_WP_	−0.94	−0.06	0.09	−0.62	0.537	−0.26	0.11
Asymmetry model	*exp*(*b*)	*b*	s.e.	*z*	*p*	*Lower*	*Upper*
R – L HV* PTE_BP_	−0.75	−0.29	0.12	−2.46	0.014*	−0.46	−0.06
R – L HV* PTE_WP_	−0.98	−0.02	0.06	−0.44	0.659	−0.14	0.08

*Note.* Table 2 presents model results, with main effects reported only for the total HV model (main effects for other models can be found in online Supplementary Tables 1 and 2). All continuous predictors were z-transformed. CES-D-10 = Center for Epidemiological Studies Depression Scale (10 items). PTE_BP_, Between-soldier effect of monthly average exposure to potentially traumatic war-zone stressors; PTE_WP_, within-soldier effect of monthly deviation from their own monthly average exposure to potentially traumatic war-zone stressors; HV, hippocampal volume (cm3), adjusted for total intracranial volume (ICV; cm3). L HV and R HV, left and right hippocampal volumes. Avg. HV, average of left and right hippocampal volumes. R – L HV, right – left hippocampal volume.

### Does total HV moderate the effect of traumatic stress on PTSD symptoms?

Total HV did not moderate the effects of within-person deviation from the average number of traumatic stressors [*b* = −0.025, *p* = 0.858 (95% CI −0.297 to 0.221)], nor the average exposure to traumatic stressors on PTSD symptoms [*b* = −0.028, *p* = 0.681 (95% CI −0.167 to 0.100)]. See Table 2 for a summary of the main effects from the model (including the direct effect of HV on PTSD symptoms).

### Does lateral HV (right or left) moderate the effect of traumatic stress on PTSD symptoms?

**Fig. 1.**
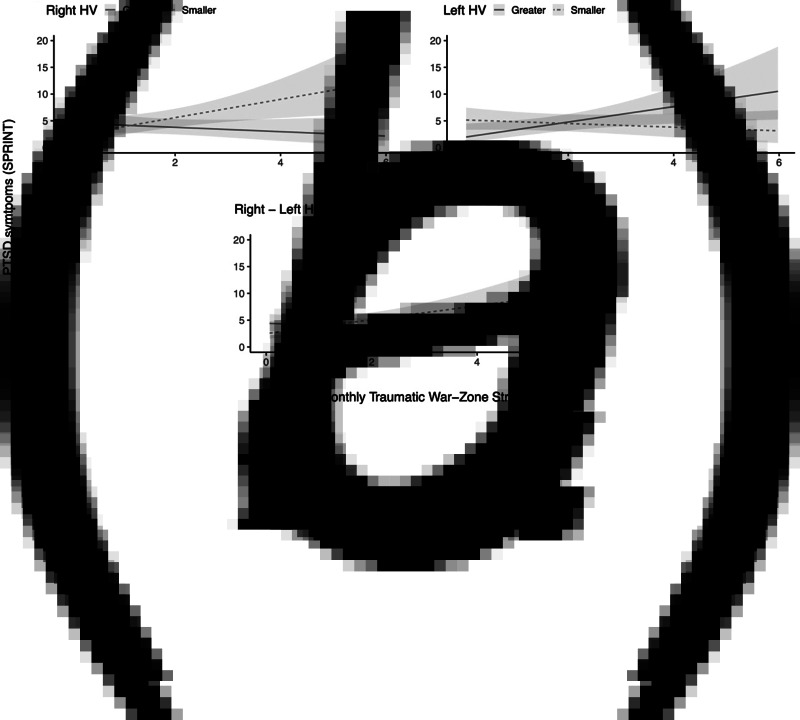
(a) Depicts that with greater number of traumatic stressors, greater right HV is associated with fewer PTSD symptoms (i.e. protective); (b) depicts that with greater number of traumatic stressors, greater left HV is associated with greater PTSD symptoms; (c) shows that when right HV is greater than left HV with greater number of traumatic stressors there are fewer PTSD symptoms, whereas the opposite is true when right HV is smaller than left HV. HV = Hippocampal Volume, ‘smaller’ = −1 standard deviation and ‘greater’ = + 1 standard deviation. ‘R > L’ reflects + 1 standard deviation from the mean lateral difference; ‘R < L’ reflects −1 standard deviation from the mean lateral difference. Values for Average Monthly Traumatic Stressor are converted from the scaled values to the approximate raw (unscaled) values. Gray bands around each regression line reflect 95% CIs.

Right *b* = −0.467, *p* = 0.023 (95% CI −0.786 to −0.013) and left *b* = 0.435, *p* = 0.024 (95% CI 0.028–0.715) HV moderated the average effect of traumatic stressors on PTSD symptoms (Fig. 1a, 1b), but not the within-person deviation from the average number of stressors *b* = −0.058, *p* = 0.537 (95% CI −0.258 to 0.113); *b* = 0.022, *p* = 0.813 (95% CI −0.164 to 0.229). Smaller right HV was associated with greater PTSD symptoms with greater average traumatic stress. However, larger left HV was associated with greater PTSD symptoms under greater traumatic stress. See online Supplementary Table S1 for a summary of the main effects from the model.

### Does HV asymmetry (right−left) moderate the effect of traumatic stress on PTSD symptoms?

Exploratory analyses were then conducted to probe the lateral asymmetry (within-person difference in right and left HV) in an exploratory analysis and found right - left asymmetry moderated the effects of average traumatic stressors on PTSD symptoms *b* = 0.287, *p* = 0.014 (95% CI −0.464 to −0.063) (Fig. 1c), although not within-person deviation *b* = 0.025, *p* = 0.657 (95% CI −0.137 to 0.077). Those with greater asymmetry (right > left) and greater average traumatic stressors reported fewer PTSD symptoms. In contrast, the opposite appeared to be true when greater asymmetry was in the opposite direction (right < left). See online Supplementary Table S2 for a summary of the main effects from the model.

## Discussion

Using a diathesis stress framework, we sought to examine the relationship between pre-deployment HV and the later in-theater emergence of monthly PTSD symptoms in response to war-zone stressors among army soldiers with no prior history of war-zone deployment. Contrary to expectations, total HV did not predict PTSD symptom emergence, nor did it moderate the effects of war-zone stressors on PTSD symptoms. However, lateral HV exerted significant effects on the relationship between war-zone stressors and PTSD symptoms. As expected, greater right HV exerted a protective effect on PTSD, whereas greater left HV was associated with greater PTSD symptom emergence. Consistent with these lateral HV findings, our post-hoc exploratory findings revealed that right > left HV asymmetry conferred reduced risk for PTSD symptom emergence even after controlling for depression and several other factors capable of influencing HV.

Prior work has shown strong support for the clinical relevance of HV asymmetry. Meta-analyses of cross-sectional studies suggest that HV asymmetry is associated with PTSD. Woon et al. (2010) found smaller right HV, but not smaller left HV in PTSD patients relative to trauma-exposed controls. A more recent meta-analysis found that smaller left but not right HV was associated with greater PTSD symptom severity (Nelson & Tumpap, 2017) and a whole brain analysis of individuals with PTSD identified smaller HV compared to trauma-exposed individuals without PTSD (Kühn & Gallinat, 2013). Importantly, right < left HV has been associated with heightened attention to trauma-related information (Hall et al., 2012), suggesting possible functional associations with risk for PTSD. Notably, prior research on HV asymmetry has been entirely cross-sectional, thus highlighting the importance of the current findings. Right > left HV asymmetry is reliably observed in healthy populations (Lucarelli et al., 2013; Woolard & Heckers, 2012), whereas right < left HV asymmetry has been linked to several neuropsychiatric disorders including schizophrenia (Oertel et al., 2010), dementia, (Geuze, Vermetten, & Bremner, 2005), and reduced cognitive ability (Woolard & Heckers, 2012). This suggests that instead of being a PTSD-specific risk factor, right < left HV asymmetry might operate as a more general marker of stress-related psychopathology.

Hippocampal atrophy has been shown to mediate the link between stress and stress-related psychopathology (Gilbertson et al., 2002; Sapolsky, 2000), whereas hippocampal growth has been shown to mediate the effects of successful treatment for PTSD (Levy-Gigi, Szabó, Kelemen, & Kéri, 2013) and depression (Warner-Schmidt & Duman, 2006). Furthermore, a range of neurotrophic factors promotes hippocampal neurogenesis (Hurley et al., 2013; Warner-Schmidt & Duman, 2006) and recent work suggests that visuo-spatial cognitive tasks (e.g. Tetris) may enhance treatment outcomes, in part due to hippocampal neurogenesis or increased synaptic plasticity (Butler et al., 2020). Future work should test whether interventions targeting hippocampal growth can protect against PTSD emergence.

### Limitations

Several limitations of the study deserve mention. First, our analyses were limited to HV measures obtained at pre-deployment and stressor exposure and stress reactions obtained during deployment in-theater. An important unanswered question is whether HV changed during deployment. Second, although there is clear value in examining hippocampal sub-regions (Chen & Etkin, 2013), we were unable to do so due to the scanner resolution. Third, although biological sex was included as a covariate, the small number of female soldiers in our sample (*n* = 14) precluded examination of biological sex differences, thus limiting the generalizability of our findings. However, while females generally expressed higher symptom levels, there were no apparent differences in the modeled relationships as a function of sex. Fourth, although the average PTSD symptom scores for the entire sample during deployment were relatively low suggesting an overall pattern of resilience, more than 25% of soldiers at some point during deployment reported a PTSD symptom score above the recommended cutoff for PTSD. Fifth, given the inherent challenges of data collection in the war-zone, missing observations were not uncommon; however, there were no patterns to missingness, our analytic approach is well suited for addressing missingness (Raudenbush & Bryk, 2002), and in-theater data were obtained from most soldiers (91%). Sixth, for similar reasons, we were unable to collect gold-standard interview measures in-theater, and thus our outcome measures are limited by the subjective nature of self-report. Finally, we applied an older version of Freesurfer (version 4.02), whereas newer versions have shown slightly less bias in the direction of overestimating HV (Schmidt et al., 2018); however, this does not preclude validly assessing individual differences in HV as a prospective stress-moderator.

## Conclusions

The present findings suggest that HV may confer risk for the emergence of trauma-related symptoms in the war zone. Importantly, we identified differential PTSD symptom emergence to traumatic-stressor exposure based on left *v.* right HV, where greater left HV was protective and smaller right HV potentiated the effect of traumatic-stressors on PTSD symptoms. Our observation that right < left HV asymmetry confers risk for the emergence of PTSD symptoms in the war zone helps explain this finding in the context of a broader asymmetry literature highlighting right < left HV asymmetry as a general risk factor for psychopathology. The findings demonstrate the utility of diathesis-stress frameworks in estimating risk conferred by neurobiological markers. Future investigations are needed to examine further the role of lateral HV and HV asymmetry as a vulnerability marker for PTSD and other stress-related disorders. Investigation of interventions that promote hippocampal neurogenesis deserves serious consideration in the prevention of PTSD and other stress-related disorders.

## Supplementary material

For supplementary material accompanying this paper visit https://doi.org/10.1017/S0033291721003548. click here to view supplementary material
