# Supplementary material for: Hippocampal volume and volume asymmetry prospectively predict PTSD symptom emergence among Iraq-deployed soldiers
Source: Psychol Med. 2021 Nov 22;53(5):1906–13. doi: 10.1017/S0033291721003548 (PMC10106285; doi:10.1017/S0033291721003548)
Supplement: Supplementary file 1 [file S0033291721003548sup001.docx]

| **Supplementary Table 1. Lateral Hippocampal Volume Model** | | | | |  | |  | | **95% CI** | | |  |
| --- | --- | --- | --- | --- | --- | --- | --- | --- | --- | --- | --- | --- |
| **Main Effects** | ***exp(b)*** | ***b*** | ***se*** | ***z*** | | ***p*** | |  | | ***Lower*** | ***Upper*** | |
| Intercept | 6.99 | 1.94 | 0.17 | 11.60 | | 0.000 | | ^***^ | | 1.63 | 2.26 | |
| Months | 1.00 | 0.00 | 0.02 | -0.09 | | 0.930 | |  | | -0.03 | 0.03 | |
| Sex (male = 0) | 1.71 | 0.54 | 0.31 | 1.72 | | 0.090 | |  | | 0.03 | 1.25 | |
| Age | -0.94 | -0.06 | 0.10 | -0.60 | | 0.550 | |  | | -0.25 | 0.14 | |
| Axis I Psychopathology (Absent = 0) | 1.50 | 0.40 | 0.21 | 1.91 | | 0.060 | |  | | 0.01 | 0.78 | |
| Prior Trauma (Absent = 0) | -0.77 | -0.26 | 0.25 | -1.05 | | 0.300 | |  | | -0.73 | 0.24 | |
| CESD | 1.76 | 0.57 | 0.08 | 7.00 | | 0.000 | | ^***^ | | 0.39 | 0.69 | |
| STAI | 1.10 | 0.10 | 0.11 | 0.89 | | 0.370 | |  | | -0.13 | 0.30 | |
| PTE_BP_ | 1.14 | 0.13 | 0.12 | 1.09 | | 0.270 | |  | | -0.12 | 0.35 | |
| PTE_WP_ | 1.16 | 0.15 | 0.06 | 2.65 | | 0.010 | | ^**^ | | 0.03 | 0.24 | |
| L HV | -0.97 | -0.03 | 0.18 | -0.16 | | 0.870 | |  | | -0.36 | 0.29 | |
| R HV | -0.84 | -0.18 | 0.17 | -1.02 | | 0.310 | |  | | -0.48 | 0.15 | |
| **Interactions** | ***exp(b)*** | ***b*** | ***se*** | ***z*** | | ***p*** | |  | |  |  | |
| L HV x PTE_BP_ | 1.54 | 0.43 | 0.19 | 2.26 | | 0.024 | | ^*^ | | 0.02 | 0.71 | |
| L HV x PTE_WP_ | 1.02 | 0.02 | 0.10 | 0.24 | | 0.813 | |  | | -0.16 | 0.23 | |
| R HV x PTE_BP_ | -0.63 | -0.47 | 0.21 | -2.27 | | 0.023 | | ^*^ | | -0.79 | -0.01 | |
| R HV x PTE_WP_ | -0.94 | -0.06 | 0.09 | -0.62 | | 0.537 | |  | | -0.26 | 0.11 | |

*Note.* Supplementary Table 1 presents model results, with main effects reported for the lateral HV model. All continuous predictors were z-transformed. CES-D-10 = Center for Epidemiological Studies Depression Scale (10 items). PTE_BP_ = Between-soldier effect of monthly average exposure to potentially traumatic war-zone stressors. PTE_WP_ = within-soldier effect of monthly deviation from their own monthly average exposure to potentially traumatic war-zone stressors. HV = hippocampal volume (cm3), adjusted for total intracranial volume (ICV; cm3). L HV and R HV = left and right hippocampal volumes.

| **Supplementary Table 2. Hippocampal Volume Asymmetry Model** | | | | | | ***95% CI*** | |
| --- | --- | --- | --- | --- | --- | --- | --- |
| **Main Effects** | ***exp(b)*** | ***b*** | ***se*** | ***z*** | ***p*** | ***Lower*** | ***Upper*** |
| Intercept | 6.99 | 1.94 | 0.17 | 11.29 | 0.000 | 1.63 | 2.25 |
| Months | 1.00 | 0.00 | 0.02 | -0.03 | 0.970 | -0.03 | 0.03 |
| Sex (male = 0) | 1.67 | 0.51 | 0.32 | 1.59 | 0.110 | -0.12 | 1.10 |
| Age | -0.94 | -0.06 | 0.10 | -0.61 | 0.540 | -0.25 | 0.12 |
| Axis I Psychopathology (Absent = 0) | 1.42 | 0.35 | 0.21 | 1.64 | 0.100 | -0.07 | 0.73 |
| Prior Trauma (Absent = 0) | -0.83 | -0.18 | 0.25 | -0.74 | 0.460 | -0.62 | 0.29 |
| CESD | 1.76 | 0.56 | 0.08 | 7.05 | 0.000 | 0.38 | 0.69 |
| STAI | 1.15 | 0.14 | 0.11 | 1.33 | 0.180 | -0.06 | 0.33 |
| PTE_BP_ | 1.14 | 0.13 | 0.12 | 1.04 | 0.300 | -0.12 | 0.35 |
| PTE_WP_ | 1.16 | 0.15 | 0.06 | 2.62 | 0.010 | 0.03 | 0.25 |
| R - L HV | -0.92 | -0.08 | 0.11 | -0.74 | 0.460 | -0.25 | 0.12 |
| **Interactions** | ***exp(b)*** | ***b*** | ***se*** | ***z*** | ***p*** | ***Lower*** | ***Upper*** |
| R - L HV* PTE_BP_ | -0.75 | -0.29 | 0.12 | -2.46 | 0.014 | -0.46 | -0.06 |
| R - L HV* PTE_WP_ | -0.98 | -0.02 | 0.06 | -0.44 | 0.659 | -0.14 | 0.08 |

*Note.* Supplementary Table 2 presents model results, with main effects reported for the asymmetry HV model. All continuous predictors were z-transformed. CES-D-10 = Center for Epidemiological Studies Depression Scale (10 items). PTE_BP_ = Between-soldier effect of monthly average exposure to potentially traumatic war-zone stressors. PTE_WP_ = within-soldier effect of monthly deviation from their own monthly average exposure to potentially traumatic war-zone stressors. HV = hippocampal volume (cm3), adjusted for total intracranial volume (ICV; cm3). R – L HV = right - left hippocampal volume.

Figure S1. A) Histogram represents the distribution of average PTSD symptom scores during deployment; B) Histogram represents the distribution of the maximum PTSD symptom score endorsed at any time during deployment.

**Overview of Supplementary Tables 3-5**

Two reviewers of earlier versions of our report requested removal of covariates to clarify the main diathesis-stress effects of interest. These effects are reported in the following tables (see Supplementary Tables 3-5). Note that all results were essentially unchanged with the removal of the covariates with the exception of a protective effect emerging for larger average HV by mitigating the impact of within-soldier deviations from average traumatic stressors on PTSD symptoms (*b* = -.14, p = .048, 95% CI: -.248, -.002; see Supplementary Table 3; cf. Table 2 in the main manuscript). While potentially interesting, we only report these results here given that inclusion of the selected covariates improved the normality of the residuals in the final models, and thus can be considered more refined and accurate estimates. Moreover, inclusion of the covariates allowed us to control for several potential confounds of the relation between hippocampal volume and PTSD symptoms (e.g., in-theater depression, prior trauma exposure, etc.).

| **Supplementary Table 3. Total HPV Model Excluding Covariates** | | | | | |  | ***95% CI*** | |
| --- | --- | --- | --- | --- | --- | --- | --- | --- |
| **Parameter** | ***exp(b)*** | ***b*** | ***se*** | ***z*** | ***p*** |  | ***Lower*** | ***Upper*** |
| Intercept | 8.23 | 2.11 | 0.14 | 15.54 | 0.000 | ^***^ | 1.877 | 2.385 |
| Months | -0.99 | -0.01 | 0.02 | -0.28 | 0.782 |  | -0.045 | 0.027 |
| PTE_BP_ | 1.03 | 0.03 | 0.13 | 0.19 | 0.846 |  | -0.214 | 0.270 |
| PTE_WP_ | 1.21 | 0.19 | 0.06 | 3.26 | 0.001 | ^**^ | 0.069 | 0.285 |
| Avg. HV | -0.85 | -0.16 | 0.13 | -1.23 | 0.220 |  | -0.413 | 0.108 |
| Avg. HV x PTE_BP_ | -0.90 | -0.11 | 0.16 | -0.68 | 0.496 |  | -0.389 | 0.200 |
| Avg. HV x PTE_WP_ | -0.87 | -0.14 | 0.07 | -1.98 | 0.048 | ^*^ | -0.248 | -0.002 |

*Note.* Supplementary Table 3 presents model results evaluating average hippocampal volume as a moderator of war-zone stressors on in-theater PTSD symptoms, excluding all covariates presented in our final models in the main manuscript. All continuous predictors were z-transformed. PTE_BP_ = Between-soldier effect of monthly average exposure to potentially traumatic war-zone stressors. PTE_WP_ = within-soldier effect of monthly deviation from their own monthly average exposure to potentially traumatic war-zone stressors. HV = hippocampal volume (cm3), adjusted for total intracranial volume (ICV; cm3). Avg. HV = total hippocampal volume.

| **Supplementary Table 4. Lateral HPV Model Excluding Covariates** | | | | | |  | ***95% CI*** | |
| --- | --- | --- | --- | --- | --- | --- | --- | --- |
| **Parameter** | ***exp(b)*** | ***b*** | ***se*** | ***z*** | ***p*** |  | ***Lower*** | ***Upper*** |
| Intercept | 7.96 | 2.07 | 0.13 | 15.91 | 0.000 | ^***^ | 1.860 | 2.345 |
| Months | 1.00 | 0.00 | 0.02 | -0.15 | 0.882 |  | -0.039 | 0.033 |
| PTE_BP_ | 1.10 | 0.09 | 0.13 | 0.71 | 0.476 |  | -0.149 | 0.330 |
| PTE_WP_ | 1.20 | 0.18 | 0.06 | 3.18 | 0.001 | ^**^ | 0.061 | 0.283 |
| R HV | -0.74 | -0.30 | 0.20 | -1.50 | 0.133 |  | -0.620 | 0.086 |
| L HV | 1.10 | 0.09 | 0.20 | 0.46 | 0.644 |  | -0.296 | 0.445 |
| L HV x PTE_BP_ | 1.56 | 0.45 | 0.22 | 2.04 | 0.042 | ^*^ | -0.017 | 0.747 |
| L HV x PTE_WP_ | 1.02 | 0.02 | 0.10 | 0.18 | 0.854 |  | -0.176 | 0.215 |
| R HV x PTE_BP_ | -0.60 | -0.52 | 0.24 | -2.14 | 0.033 | ^*^ | -0.870 | -0.008 |
| R HV x PTE_WP_ | -0.87 | -0.13 | 0.10 | -1.38 | 0.169 |  | -0.317 | 0.067 |

*Note.* Supplementary Table 4 presents model results evaluating left and right hippocampal volume as moderators of war-zone stressors on in-theater PTSD symptoms, excluding all covariates presented in our final models in the main manuscript. All continuous predictors were z-transformed. PTE_BP_ = Between-soldier effect of monthly average exposure to potentially traumatic war-zone stressors. PTE_WP_ = within-soldier effect of monthly deviation from their own monthly average exposure to potentially traumatic war-zone stressors. HV = hippocampal volume (cm3), adjusted for total intracranial volume (ICV; cm3). L HV = left hippocampal volume. R HV = right hippocampal volume.

| **Supplementary Table 5. HPV Asymmetry Model Excluding Covariates** | | | | | | | ***95% CI*** | |
| --- | --- | --- | --- | --- | --- | --- | --- | --- |
| **Parameter** | ***exp(b)*** | ***b*** | ***se*** | ***z*** | ***p*** |  | ***Lower*** | ***Upper*** |
| Intercepts | 7.75 | 2.05 | 0.13 | 15.72 | 0.000 | ^***^ | 1.824 | 2.304 |
| Months | 1.00 | 0.00 | 0.02 | -0.09 | 0.933 |  | -0.037 | 0.036 |
| R - L HV | -0.86 | -0.15 | 0.12 | -1.29 | 0.199 |  | -0.343 | 0.083 |
| PTE_BP_ | 1.10 | 0.09 | 0.13 | 0.69 | 0.493 |  | -0.145 | 0.349 |
| PTE_WP_ | 1.19 | 0.17 | 0.06 | 3.01 | 0.003 | ^**^ | 0.056 | 0.281 |
| R - L HV x PTE_BP_ | -0.73 | -0.31 | 0.14 | -2.30 | 0.022 | ^*^ | -0.503 | -0.015 |
| R - L HV x PTE_WP_ | -0.94 | -0.06 | 0.06 | -1.06 | 0.288 |  | -0.175 | 0.050 |

*Note.* Supplementary Table 5 presents model results evaluating right – left hippocampal volume asymmetry as a moderator of war-zone stressors on in-theater PTSD symptoms, excluding all covariates presented in our final models in the main manuscript. All continuous predictors were z-transformed. PTE_BP_ = Between-soldier effect of monthly average exposure to potentially traumatic war-zone stressors. PTE_WP_ = within-soldier effect of monthly deviation from their own monthly average exposure to potentially traumatic war-zone stressors. HV = hippocampal volume (cm3), adjusted for total intracranial volume (ICV; cm3). L HV = left hippocampal volume. R HV = right hippocampal volume. R - L HV = right – left hippocampal volume asymmetry.

| **Supplementary Table 6. Correlation Matrix Including All Modeled Variables** | | | | | | | |
| --- | --- | --- | --- | --- | --- | --- | --- |
| **Variable** | **1** | **2** | **3** | **4** | **5** | **6** | **7** |
| 1. Sex (male = 0) | 1.00 | - | - | - | - | - | - |
| 2. Age | -0.05 | 1.00 | - | - | - | - | - |
| 3. Prior trauma | 0.08 | 0.13 | 1.00 | - | - | - | - |
| 4. STAI | 0.18 | -0.21 | -0.01 | 1.00 | - | - | - |
| 5. Axis I Psychopathology (Absent = 0) | -0.02 | -0.29 | -0.08 | 0.40 | 1.00 | - | - |
| 6. L HV | -0.08 | -0.07 | -0.26 | -0.15 | 0.26 | 1.00 | - |
| 7. R HV | -0.05 | 0.10 | -0.18 | -0.08 | 0.15 | 0.73 | 1.00 |
| 8. Avg. HV | 0.30 | 0.00 | -0.21 | -0.07 | 0.18 | 0.83 | 0.86 |
| 9. R - L HV | 0.02 | 0.24 | 0.05 | 0.06 | -0.13 | -0.18 | 0.54 |
| 10. Months | 0.04 | 0.18 | 0.12 | 0.04 | 0.03 | -0.22 | -0.05 |
| 11. PTE_BP_ | -0.58 | -0.34 | 0.15 | 0.00 | 0.06 | 0.10 | -0.05 |
| 12. PTE_WP_ | -0.06 | 0.00 | -0.04 | 0.00 | -0.06 | 0.04 | 0.03 |
| 13. CES-D-10 | 0.29 | 0.15 | -0.13 | -0.01 | -0.03 | 0.01 | -0.03 |
| 14. SPRINT | 0.29 | -0.12 | -0.15 | 0.08 | 0.27 | -0.07 | -0.10 |
| **Variable** | **8** | **9** | **10** | **11** | **12** | **13** | **14** |
| 1. Sex (male = 0) | - | - | - | - | - | - | - |
| 2. Age | - | - | - | - | - | - | - |
| 3. Prior trauma | - | - | - | - | - | - | - |
| 4. STAI | - | - | - | - | - | - | - |
| 5. Axis I Psychopathology (Absent = 0) | - | - | - | - | - | - | - |
| 6. L HV | - | - | - | - | - | - | - |
| 7. R HV | - | - | - | - | - | - | - |
| 8. Avg. HV | 1.00 | - | - | - | - | - | - |
| 9. R - L HV | 0.22 | 1.00 | - | - | - | - | - |
| 10. Months | -0.12 | 0.20 | 1.00 | - | - | - | - |
| 11. PTE_BP_ | -0.09 | -0.19 | -0.20 | 1.00 | - | - | - |
| 12. PTE_WP_ | 0.02 | 0.00 | -0.11 | 0.01 | 1.00 | - | - |
| 13. CES-D-10 | 0.05 | -0.05 | 0.00 | -0.11 | 0.08 | 1.00 | - |
| 14. SPRINT | -0.01 | -0.05 | 0.04 | 0.01 | 0.05 | 0.48 | 1.00 |

*Note.* Supplementary Table 6 presents Pearson, polychoric, and polyserial correlations, as appropriate, for all modeled variables. STAI = State-Trait Anxiety Inventory. PTE_BP_ = Between-soldier effect of monthly average exposure to potentially traumatic war-zone stressors. PTE_WP_ = within-soldier effect of monthly deviation from their own monthly average exposure to potentially traumatic war-zone stressors. HV = hippocampal volume (cm3), adjusted for total intracranial volume (ICV; cm3). L HV = left hippocampal volume. R HV = right hippocampal volume. R - L HV = right – left hippocampal volume asymmetry. CES-D-10 = Center for Epidemiological Studies Depression Scale (10 items), administered monthly during deployment. SPRINT = Short Post-Traumatic Stress Disorder Rating Interview, administered monthly during deployment.
